# In Situ Construction of 2D/2D g-C_3_N_4_/rGO Hybrid Photocatalysts for Efficient Ciprofloxacin Degradation

**DOI:** 10.3390/nano15211641

**Published:** 2025-10-28

**Authors:** Mengyao Wang, Yong Li, Rui Li, Yali Zhang, Deyun Yue, Shihao Zhao, Maosong Chen, Haojie Song

**Affiliations:** 1 School of Materials Science and Engineering, Shaanxi University of Science and Technology, Xi’an 710021, China; 15518195575@163.com (M.W.); lirui202207@163.com (R.L.); 14795660208@163.com (Y.Z.); 18092840228@163.com (D.Y.); 15319925655@163.com (S.Z.); 18395168606@163.com (M.C.); 2 Shaanxi Key Laboratory of Green Preparation and Functionalization for Inorganic Materials, Shaanxi Laboratory of Advanced Materials, Xi’an 710021, China

**Keywords:** photocatalytic degradation, solid-phase synthesis, 2D/2D composite photocatalysts

## Abstract

Insufficient harvesting of visible photons, limited adsorption, and fast recombination of photogenerated electron-hole pairs restrict the application of graphitic carbon nitride (g-C_3_N_4_). Here, we propose a straightforward solid-phase synthesis method for fabricating 2D/2D graphitic carbon nitride/reduced graphene oxide (SCN/GR) hybrid photocatalysts. The synthesis process involves the thermal condensation of three precursors: dicyandiamide (as the g-C_3_N_4_ source), NH_4_Cl (as a pore-forming agent), and graphene oxide (GO, which is in situ reduced to rGO during thermal treatment). The incorporation of reduced graphene oxide (rGO) into the g-C_3_N_4_ matrix not only narrows the bandgap of the material but also expedites the separation of photogenerated carriers. The photocatalytic activity of the SCN/GR hybrid was systematically evaluated by degrading ciprofloxacin in aqueous solution under different light conditions. The results demonstrated remarkable degradation efficiency: 72% removal within 1 h under full-spectrum light, 81% under UV light, and 52% under visible light. Notably, the introduction of rGO significantly improved the visible light absorption capacity of g-C_3_N_4_. Additionally, SCN/GR exhibits exceptional cyclic stability, maintaining its structural integrity and photocatalytic properties unchanged across five successive degradation cycles. This study offers a simple yet effective pathway to synthesize 2D/2D composite photocatalysts, which hold significant promise for practical applications in water treatment processes.

## 1. Introduction

Water is a vital natural resource that covers a substantial portion of the Earth’s surface, yet a mere fraction of freshwater is accessible for human use [1]. Water resource scarcity is compounded by global natural changes and the escalating consumption demands driven by human activities [2,3]. In recent years, the extensive use of antibiotics globally has significantly enhanced the treatment of infectious diseases and agricultural productivity [4]. However, a significant fraction of these antibiotics escapes complete metabolism and is subsequently discharged into aquatic environments [5]. The physical and chemical inertness of antibiotics poses a substantial challenge for their removal from water bodies, potentially heightening aquatic toxicity levels [6]. The presence of antibiotics in the environment can lead to detrimental effects, including genetic exchange and the development of antibiotic-resistant bacteria [7,8].

To address the environmental challenges at hand, researchers have proactively investigated and developed a range of treatment technologies, including adsorption, membrane filtration, electrocatalysis, biodegradation, and photocatalysis [9,10,11,12]. Photocatalysis stands out as an advanced oxidation process, offering a cost-effective means to decompose toxic and noxious pollutants [13]. g-C_3_N_4_ has garnered significant attention as a promising inorganic semiconductor photocatalyst, prized for its stability, non-toxicity, and tunable energy band structure, which collectively render it well-suited for water environmental remediation [14,15,16,17]. Despite these benefits, g-C_3_N_4_ faces limitations due to its small specific surface area, limited utilization of visible light, and high rate of electron-hole pair recombination, which hinder its photocatalytic efficacy [18,19,20]. To enhance g-C_3_N_4_’s activity, various strategies have been devised, including morphological modification, heterojunction construction, defect engineering, and doping [21,22,23,24,25,26].

Two-dimensional (2D) materials, with their planar structures, offer an optimal platform for maximizing photocatalytic performance through the exploitation of their unique dimensionality [27]. The ultrathin nature of 2D materials minimizes lattice mismatch, facilitating the formation of intimate contact interfaces between the 2D photocatalysts and reducing barriers to electron transfer [28,29]. The integration of 2D co-catalysts can increase the active sites or tailor their properties for specific photocatalytic reactions [30]. Graphene, a two-dimensional carbon material, is renowned for its excellent carrier mobility and exceptional electrical conductivity [31]. For practical applications, graphene is commonly used in the form of reduced graphene oxide, activated graphene, doped graphene, graphene/metal oxide composites, or graphene/polymer composites [32]. In recent years, the combination of semiconductors with graphene has been a subject of intensive research. Zhang et al. [33] reported that reduced graphene oxide (rGO) enhanced the adsorption properties of FeS_2_. The interface between the photocatalyst and graphene not only accelerated the charge transfer rate but also increased the number of active centers, with its oxygen functional groups facilitating the adsorption of pollutants [34]. The broad-spectrum light absorption property of graphene aids in enhancing the utilization of visible light by the photocatalyst. Consequently, devising a simple and eco-friendly method to composite g-C_3_N_4_ with graphene represents an effective approach to address the challenge of enhancing g-C_3_N_4_’s photocatalytic activity.

In this study, we introduce a straightforward and eco-friendly method for the synthesis of visible light-activated rGO/g-C_3_N_4_ composite photocatalysts. Irregular thin layers of g-C_3_N_4_ nanosheets were grown in situ on rGO nanosheets through the thermal condensation of dicyandiamide, NH_4_Cl, and graphene oxide. The incorporation of graphene nanosheets generated additional active sites, which improved adsorption and photocatalytic degradation capabilities. The intimate bonding between g-C_3_N_4_ and rGO fostered a strong interfacial effect, accelerating the separation of photogenerated carriers. The presence of graphene enhanced the light absorption of g-C_3_N_4_ in the visible region, thereby boosting the photocatalytic performance of the SCN/GR composite under visible light irradiation. The SCN/GR composite exhibited higher photocatalytic activity than bulk g-C_3_N_4_ and g-C_3_N_4_ nanosheets under full-spectrum solar, UV, and visible light conditions. Furthermore, SCN/GR demonstrated excellent cycling stability, making it a promising candidate for the efficient treatment of antibiotic-containing wastewater under visible light exposure.

## 2. Experimental Sections

### 2.1. Preparation of Bulk g-C_3_N_4_

First, 2 g of dicyandiamide was placed into an alumina crucible with a cover and heated at 550 °C in Ar for 4 h, with a heating rate of 5 °C/min. The resultant material was ground into powder and denoted as BCN.

### 2.2. Preparation of g-C_3_N_4_ Nanosheets

Equal mass ratios of dicyandiamide and NH_4_Cl are mixed thoroughly and transferred to a covered alumina crucible, which is then heated at 550 °C in Ar for 4 h, and a heating rate of 5 °C/min. The resultant materials were denoted as SCN.

### 2.3. Preparation of Reduced Graphene Oxide/Bulk g-C_3_N_4_

2 g of dicyandiamide was mixed with 2 mL of 10 mg/mL GO dispersion to form a paste mixture, which was later collected in a covered alumina crucible. The mixture was heated at 550 °C in Ar for 4 h, with a heating rate of 5 °C/min. The resultant materials were denoted as BCN/GR.

### 2.4. Preparation of Reduced Graphene Oxide/g-C_3_N_4_ Nanosheets (SCN/GR)

Equal mass ratios of dicyandiamide and NH_4_Cl were thoroughly mixed, followed by the addition of 2 mL of 10 mg/mL GO dispersion to form a paste mixture. The mixture was then collected in a covered alumina crucible. It was heated at 550 °C in Ar for 4 h, with a heating rate of 5 °C/min. The resultant products were designated as SCN/GR.

### 2.5. Photoelectrochemical Measurements

To evaluate the charge transfer properties of the samples, photoelectrochemical tests were carried out using a three-electrode system, in which Ag/AgCl was used as the reference electrode and a platinum sheet as the counter electrode. The slurry was prepared by dispersing the photocatalyst with Nafion solution in ethanol. The slurry of photocatalyst was coated on an indium tin oxide glass substrate and then dried as the working electrode. The electrolyte solution was a Na_2_SO_4_ aqueous solution (100 mL, 0.1 M), and a xenon lamp was used to simulate visible light irradiation.

### 2.6. Photocatalytic Tests

The photocatalytic performance was evaluated by degrading a variety of target pollutants (ciprofloxacin, sulfamethoxazole, rhodamine B, and methylene blue, etc.) at room temperature. 30 mg of photocatalyst was added to 50 mL of 20 mg/L target pollutant solution and reacted in the dark for 1 h. After adsorption equilibrium, photocatalysis degradation was performed with a xenon lamp. 2 mL of the dispersion was pipetted and centrifuged at intervals of 10 min, and the supernatant was collected, and the absorbance was measured by a UV-visible spectrophotometer.

## 3. Results and Discussion

### 3.1. Materials Characterization

**Figure 1:**
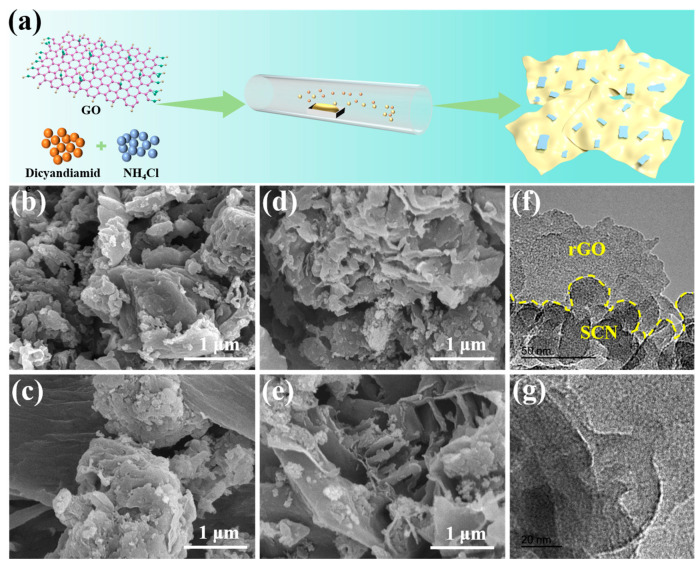
(**a**) Schematic for the preparation of SCN/GR. SEM images of BCN (**b**), BCN/GR (**c**), SCN (**d**), and SCN/GR (**e**). (**f**,**g**) TEM images of SCN/GR.

A one-step in situ synthesis method was employed to fabricate the g-C_3_N_4_/rGO composite photocatalyst. As depicted in Figure 1a, NH_4_Cl served as a blowing agent, decomposing under high-temperature conditions to yield NH_3_(g) and HCl(g), which facilitated the formation of g-C_3_N_4_ nanosheets with a significantly reduced thickness. During calcination, GO was transformed into rGO in the presence of Ar, functioning as a template for the self-assembly of g-C_3_N_4_ into a 2D/2D layered structure. This process suppressed the self-stacking tendency of graphene, thereby generating more active sites and improving charge transfer properties.

The morphology of the photocatalyst was characterized using SEM and TEM. As shown in Figure 1b, the BCN structure exhibited an irregular and aggregated arrangement, which is not conducive to photocatalytic degradation. The layered architecture of the BCN/GR composite is illustrated in Figure 1c. However, pronounced stacking was observed in both BCN and rGO, hindering the separation of photogenerated electron-holes. The introduction of NH_4_Cl resulted in a significant reduction in the lamellar thickness of the SCN, which increased the active sites and specific surface area, thereby enhancing reactivity (Figure 1d). The intimate interaction between rGO flakes and the 2D SCN structure in Figure 1e suggests the formation of an interfacial boundary. This interfacial interaction mitigated the self-stacking tendencies of both SCN and rGO, leading to a composite structure that optimized charge transfer at the SCN/rGO interface. As a result, the SCN/GR composite demonstrated enhanced photocatalytic activity. The TEM images in Figure 1f,g revealed a smooth rGO layer and uniformly dispersed SCN nanosheets with a typical small, thin, and irregular 2D morphology atop the rGO film. The absence of aggregation in either SCN or rGO indicated that the incorporation of SCN could mitigate the tendency of the graphene layer to curl. Therefore, the SCN/GR composite offered improved charge transfer rates and superior photodegradation performance.

**Figure 2:**
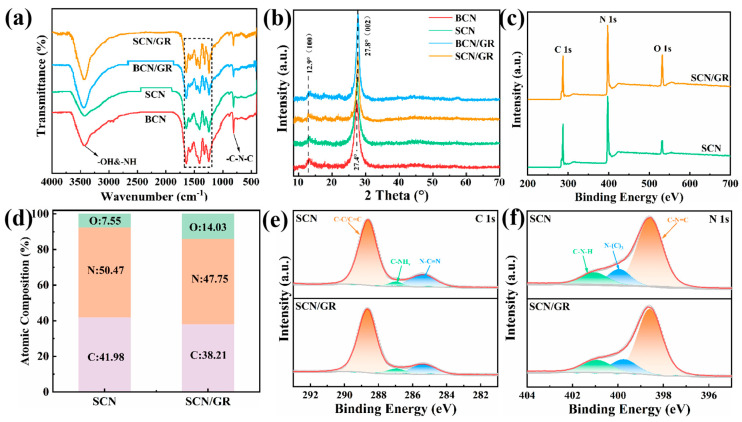
(**a**) FT−IR spectra. (**b**) XRD patterns. (**c**) XPS survey spectra of SCN and SCN/GR. (**d**) The element content of SCN and SCN/GR. (**e**) C 1s spectra of SCN and SCN/GR. (**f**) N 1s spectra of SCN and SCN/GR.

FT-IR spectroscopy was employed to probe the surface functional groups. As depicted in Figure 2a, due to the low content of rGO, only the characteristic peaks of SCN were observed in the spectra of SCN/GR composites. The overall patterns of the samples were similar, indicating that the presence of rGO did not significantly alter the core structure of the SCN framework. The strong bands between 1200 and 1700 cm^−1^ corresponded to stretching vibrations characteristic of aromatic CN heterocycles. Specifically, the peaks at 1648, 1470, and 1410 cm^−1^ are attributed to the stretching vibrations of heptazine-derived repeating units. The peaks at 1327 and 1240 cm^−1^ correspond to the out-of-plane bending vibrations that are characteristic of heptazine rings. Additionally, the sharp peak around 810 cm^−1^ originates from the typical stretching vibration of the triazine unit. The broad peak between 3000–3600 cm^−1^ was attributed to O-H and N-H stretching [35,36]. The absence of C-O or C=O bonds in SCN/GR suggested that most oxygen functional groups on GO were removed via thermal reduction.

The crystallinity and phase purity of the BCN, SCN, BCN/GR, and SCN/GR samples were assessed via XRD analysis. Figure 2b presents the XRD patterns for each sample, which exhibit two distinct reflection peaks. The peaks located at 12.9° and 27.4° are the most prominent and clearly indicate the formation of a graphitic crystal structure in g-C_3_N_4_. The peak at 12.9° is attributed to the (100) crystal plane, representing a structural rearrangement pattern between the 3 s-triazine (heptazine) layers, while the peak at 27.4° corresponds to the (002) plane, reflecting the periodic stacking of aromatic rings [37,38]. Notably, the XRD spectra of the BCN/GR and SCN/GR catalysts are similar to those of BCN and SCN, albeit with a peak shift observed. The peak at 2θ = 27.8° in BCN/GR and SCN/GR suggests effective coupling between g-C_3_N_4_ and rGO. It is noteworthy that characteristic peaks of GO and rGO are not observed in the BCN/GR and SCN/GR spectra, which may be due to GO disrupting the regular stacking of graphene during thermal reduction.

The elemental composition of the SCN and SCN/GR samples was determined using X-ray photoelectron spectroscopy (XPS), with the results depicted in Figure 2c. The SCN sample contained 41.98% carbon, 50.47% nitrogen, and 7.55% oxygen (Figure 2d). The increase in oxygen content to 14.03% in the SCN/GR is attributed to the influence of oxygen-containing functional groups from GO. Studies have shown that quinone groups decompose between 500 and 900 °C, with oxygen groups being completely removed at ~1100 °C. The C 1s peak in Figure 2e consists of three components, corresponding to sp2-hybridized carbon, residual NH_x_ on the edge of the heptazine unit, and C-C bonds in the carbon-nitrogen aromatic framework. The N 1s peak (Figure 2f) can be divided into three peaks at 401.1, 399.9, and 398.6 eV. The peak at 398.6 eV is associated with sp2-hybridized nitrogen bonded to two carbon atoms (C-N=C), which is the primary nitrogen species in g-C_3_N_4_ with a 3 s-triazine structure [39]. The other peaks at 401.1 and 399.9 eV are ascribed to quaternary nitrogen (N with three carbon atoms, N-(C)_3_) and amino (C-N-H) functionalities, respectively [40].

### 3.2. Physicochemical Properties of Photocatalysts

**Figure 3:**
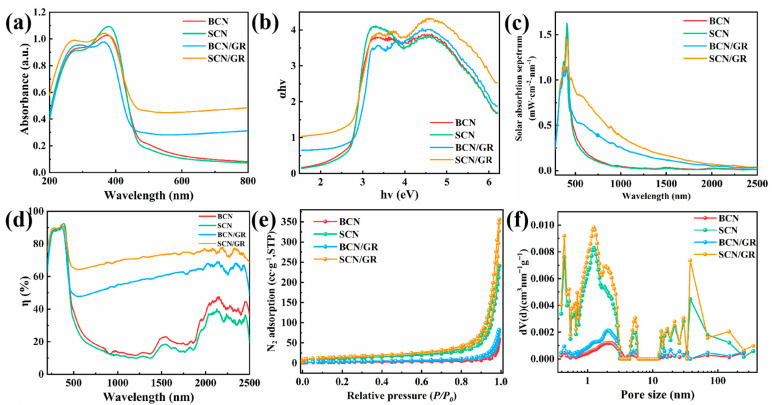
(**a**) DRS spectra. (**b**) Tauc plots. (**c**) Absorption power spectra. (**d**) Light harvesting efficiency. (**e**) Adsorption–desorption curves. (**f**) Pore size distribution.

Optical properties of the photocatalysts were characterized using a UV-visible spectrophotometer. BCN and SCN displayed similar absorption spectra, with absorption edges at 480 nm and 472 nm, respectively (Figure 3a). The similarity in absorption curves and edges indicated that the addition of NH_4_Cl had a minimal impact on BCN’s light absorption capacity. Research indicates that g-C_3_N_4_ is a direct-bandgap semiconductor. The absorption coefficient α of a direct-bandgap semiconductor is related to the photon energyhν by the equation αhν = B(hν − Eg)^1/2^. Squaring both sides of αhν = B(hν − Eg)^1/2^ yields (αhν)^2^ = B^2^(hν − Eg). Plot the Tauc diagram with (αhν)^2^ on the vertical axis and hν on the horizontal axis. In the Tauc plot, the linear portion of the curve is extrapolated to the vertical coordinate of 0. The horizontal coordinate value corresponding to this point represents the bandgap width (Eg) (i.e., the slope’s intercept). Therefore, BCN and SCN exhibited similar band gaps of 2.68 and 2.69 eV, respectively (Figure 3b). The incorporation of rGO, which is black in color, enhances visible light absorption. Consequently, the composite photocatalyst exhibited an extended light absorption range across the entire wavelength spectrum following rGO incorporation, which is crucial for the efficient utilization of solar energy. However, it was observed that the light absorption of BCN/GR and SCN/GR exhibited contrasting trends following the addition of rGO. The blue shift in the absorption edge of BCN upon rGO addition is attributed to the aggregation of nanosheets. Although rGO extends the light absorption range, the absorption edge shifted from 480 nm to 468 nm, and the band gap increased. Conversely, when rGO was added to SCN, the formation of nanosheets and increased quantum effects led to a red shift in the absorption edge from 472 nm to 501 nm, and the band gap narrowed [41]. These results suggest that the successful introduction of rGO and NH_4_Cl can increase the light absorption in the visible region, which leads to the production of more photogenerated carriers under visible light irradiation and further improves the quantum efficiency. In order to investigate the utilization of solar energy by the photocatalysts, the light absorption of the four photocatalysts under simulated sunlight (AM 1.5G) was investigated. The absorbed power spectra of solar energy (Figure 3c) showed that BCN/GR and SCN/GR exhibited obvious enhancement of light absorption and significant utilization of solar energy. Among these, the SCN/GR exhibited higher solar energy absorption and extremely high utilization of solar energy. To further explore light utilization by the photocatalysts, the light capture efficiency was investigated (Figure 3d). The results showed that the light capture efficiency of SCN/GR was higher than 60% in the region of 200–2500 nm, and the utilization of light was high in all spectral ranges. In summary, the light absorption range and utilization of SCN/GR were enhanced.

The pore structure and specific surface area of the samples were characterized by the N_2_ adsorption–desorption test. As shown in Figure 3e, the specific surface areas of BSN, SCN, BCN/GR, and SCN/GR were 7.89 m^2^ g^−1^, 40.56 m^2^ g^−1^, 13.38 m^2^ g^−1,^ and 56.41 m^2^ g^−1^, respectively (Table S1). Specifically, SCN/GR exhibits a markedly larger specific surface area than BSN, SCN, or BCN/GR. This gain stems from a synergistic interplay between graphene and NH_4_Cl that templates an open, hierarchically porous network. Interestingly, the specific surface area of BCN/GR catalysts was slightly higher than that of BCN, indicating that the presence of rGO increased the specific surface area of BCN to a certain extent. The pore size distribution characteristics of SCN and SCN/GR were similar, and micropores, mesopores, and macropores existed in the samples at the same time, which showed different levels of pore structure, which is conducive to the promotion of surface photocatalytic reaction (Figure 3f, Table S1). The above results indicated that the introduction of graphene and NH_4_Cl led to an increase in both specific surface area and pore volume, which was conducive to the improvement of photocatalytic activity.

**Figure 4:**
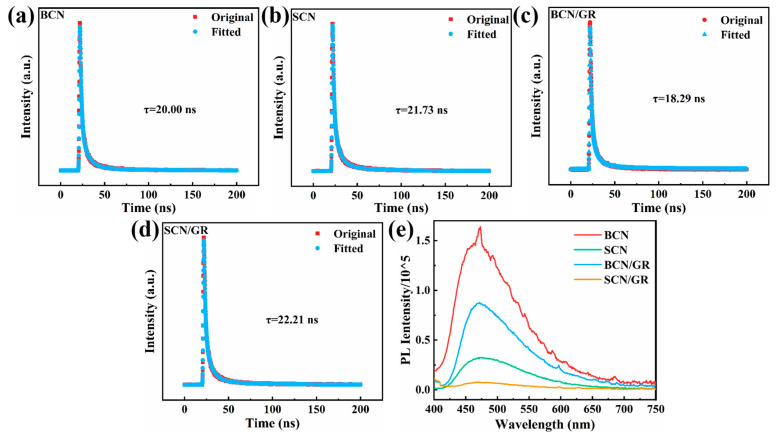
(**a**–**d**) Time Resolved Photoluminescence spectroscopy and (**e**) PL spectra of BCN, SCN, BCN/GR, and SCN/GR.

Photoluminescence and time-resolved photoluminescence spectroscopy are used to analyze the recombination of photogenerated carriers. Photoluminescence is characterized by the emission of light after the material absorbs a photon. The Time-resolved photoluminescence spectra of the four catalysts are fitted in Figure 4a–d. The average fluorescence lifetime of SCN/GR is 22.21 ns, which is higher than BCN (20.00 ns), SCN (21.73 ns), and BCN/GR (18.29 ns). The higher average lifetime of SCN/GR indicates that the increase in excitation lifetime of the photogenerated charge carriers is conducive to the dissociative charge and active particle binding. Under typical circumstances, low PL intensity represents low charge complexation. From Figure 4e, it can be seen that the intensity of BCN is the highest among all catalysts, while the intensity of SCN/GR is the lowest, which proves that the complexation of SCN and rGO contributes to the enhancement of photocatalytic performance. This is due to the transfer of electrons to graphene sheets after jumping to the conduction band, which enhances the photogenerated electron-hole separation efficiency. As a result, the graphene sheet becomes the separation center of photogenerated electrons and holes because graphene is a good electron acceptor material to transfer electrons efficiently. As a result, the photogenerated carriers generated by SCN/GR exhibit higher stability and can initiate redox reactions before the occurrence of recombination. The increase in photocatalytic activity brought about by SCN/GR is attributed to the improved separation of photogenerated electrons and holes. Both the high quantity of active sites in nanostructures and the direct, fast transfer routes of carriers contribute to a marked improvement in carrier separation.

**Figure 5:**
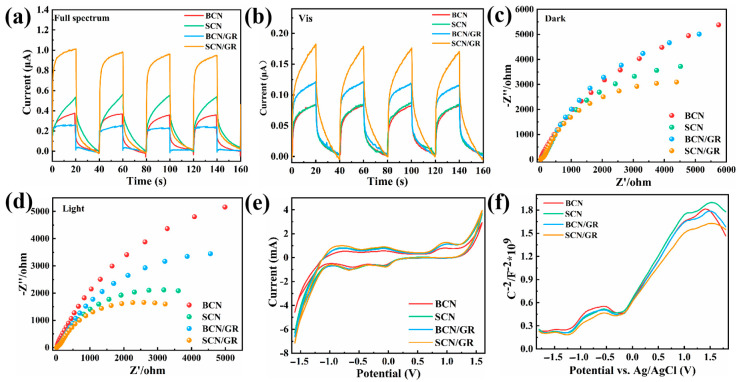
Transient photocurrent in (**a**) full spectrum and (**b**) visible light diagram. Electrochemical impedance diagram in (**c**) dark and (**d**) light conditions. (**e**) Cyclic voltammetry curves. (**f**) Mott-Schottky plots of various samples.

Combining photoluminescence spectroscopy with photoelectrochemical detection allows for the simultaneous determination of photogenerated carrier separation and photocatalytic properties of photocatalysts. To investigate the charge transfer properties, EIS and photocurrent measurements were performed on the prepared samples. Figure 5a,b show the photocurrents of the photocatalysts under intermittent full-spectrum sunlight as well as visible light irradiation. For all samples, the photocurrent intensity increases dramatically when the light is switched on. It can be seen that the current intensity of SCN/GR is significantly higher than that of BCN, SCN, and BCN/GR under both full-spectrum and visible-light irradiation. The combination of rGO with g-C_3_N_4_ improves the photocatalytic activity because of its lamellar structure and the prominent electronic nature that hinders the binding of electrons to holes [42]. The reduced photocurrent of BCN/GR under full-spectrum irradiation could be due to the severe lamellar stacking, which leads to relatively rapid complexation of electron-hole pairs. However, the photocurrent values of SCN/GR were still 2.5 times higher than those of BCN and SCN, and 4 times higher than those of BCN/GR, indicating the superior photocatalytic activity. Under visible light irradiation, BCN/GR showed a higher photocurrent than BCN and SCN, attributed to enhanced visible-light absorption. In addition, the transient photocurrent response of SCN/GR remained stable after several on/off cycles, indicating its good photostability. The charge transfer kinetics at the interface under dark and light conditions were further evaluated using the EIS method. In Figure 5c,d, the radius of SCN/GR is the smallest under both dark and light conditions, indicating that its electron transfer kinetics are faster than those of BCN, SCN, and BCN/GR. These results further demonstrate that the SCN/GR heterojunction system is favorable for the efficient separation and migration of photogenerated carriers. In addition, the radius of the SCN/GR is significantly larger in the dark than in the light, which suggests that the resistance is smaller in the light, permitting a large amount of charge transport (Figure S1a). It also means that the electron transport rate is faster, indicating that more photogenerated carriers are produced. The above changes indicate that the introduction of graphene does improve the carrier transfer in g-C_3_N_4_ and that graphene can be used as a new transfer pathway for photogenerated carriers.

The redox ability of the photocatalysts was tested using linear scanning voltammetry and cyclic voltammetry. As can be seen in Figure S1b, a distinct oxidation peak belongs to the oxygen reaction at around 1.04 V. Obviously, the oxidation peaks of SCN/GR photocatalysts are stronger than those of BCN, SCN, and BCN/GR photocatalysts, which show a stronger oxidation capacity. The CV curves are shown in Figure 5e. A pair of clear characteristic redox peaks was observed in the range of −1.6~1.6 V, which is consistent with the LSV curve. Notably, the area integrals of SCN/GR within the CV curves were larger than those of pure BCN, SCN, and BCN/GR, indicating that the prepared SCN/GR photocatalysts possessed a higher redox capacity. The flat-band potentials of all samples were determined by selecting Mott-Schottky (Figure 5f). The flat-band positions of BCN, SCN, BCN/GR, and SCN/GR were −0.56, −0.58, −0.55, and −0.64 V (relative to Ag/AgCl), respectively. This is due to the quantum confinement effect induced by the hierarchical micro-nano-structure, leading to the shift in the CB of SCN/GR towards negative values.

### 3.3. Photocatalytic Performance

**Figure 6:**
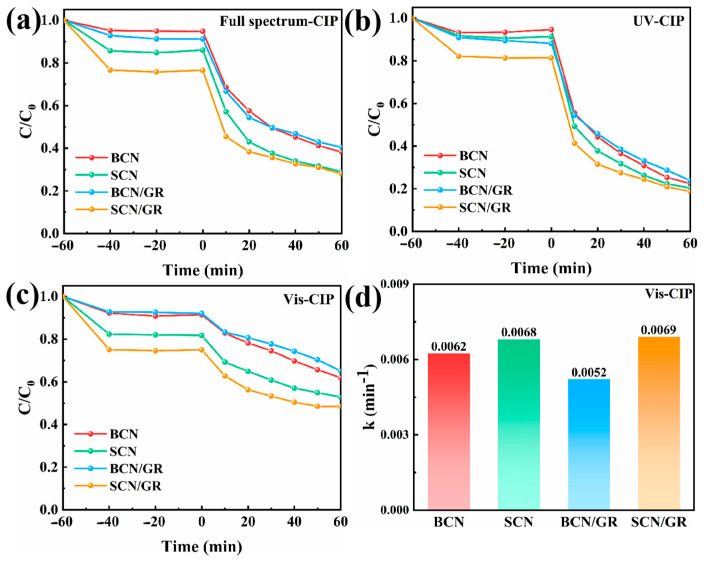
Photodegradation curves on different photocatalysts of CIP under (**a**) full spectrum, (**b**) UV, and (**c**) visible light. (**d**) Corresponding apparent rate constants for CIP under visible light.

Figure 6a–c show the degradation effects of the photocatalysts on CIP under full-spectrum sunlight, UV light, and visible light irradiation. It can be seen that the adsorption effects of the photocatalysts on CIP under different light irradiations showed similar trends. The adsorption of CIP by BCN and BCN/GR was small, with less than 10% of CIP adsorbed in 60 min, which was consistent with the results of SEM characterization, further proving that the addition of rGO alone could not improve the self-stacking phenomenon of g-C_3_N_4_. The adsorption performance of SCN/GR on CIP was significantly improved after the introduction of NH_4_Cl and rGO. When CIP was dark adsorbed on SCN/GR for 60 min, the removal rate of CIP was about 25%. The results confirmed that it has a 2D/2D structure that favors adsorption. On the other hand, it can be seen that the adsorption capacity is basically saturated after 20 min of adsorption, which means that the sample, serving as an efficient adsorbent, enables the rapid adsorption of pollutants. Under different light irradiation, both SCN/GR showed the highest degradation performance. And it showed obvious photocatalytic performance enhancement under visible light, which further proved that the introduction of rGO successfully broadened the light absorption range of SCN. Obviously, the graphene-containing SCN/GR was largely superior to the pristine SCN. The enhanced visible-light photocatalytic activity can be attributed to the critical role of graphene in promoting the conduction of photoinduced electrons. However, the tests revealed no significant change in the photocatalytic performance after the introduction of rGO into BCN. This was attributed to the light shielding effect produced by rGO in the reactor after the composite hindered the photocatalytic degradation reaction, thus weakening the photocatalytic performance of the photocatalyst [14]. In summary, the introduction of NH_4_Cl and rGO contributes to the enhancement of photocatalytic activity. The unique 2D/2D sparse structure of SCN/GR not only creates abundant active sites and facilitates the homogeneous dispersion of nanoparticles, but also enhances the chemical and structural stability of the nanoparticles. Due to the excellent electron trapping and light absorption properties of rGO, the electron mobility rate and the separation probability of electron-hole pairs were significantly increased. These results synergistically modified the photocatalytic performance of SCN/GR composites.

**Figure 7:**
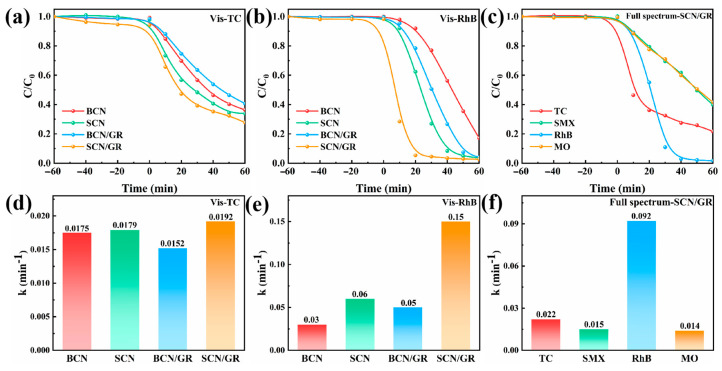
Photodegradation curves of (**a**) TC (**b**) RhB under visible light. (**c**) Photodegradation curves of organic pollutants on SCN/GR under full spectrum. (**d**–**f**) Corresponding apparent rate constants.

In order to further demonstrate the visible-light-enhanced photocatalytic effect of SCN/GR, the photocatalytic performance of SCBN/GR under visible light was investigated more deeply. As shown in Figure 7a,b, SCN/GR exhibited the highest degradation performance for TC and RhB under visible light (TC, 74%; RhB, 98%). The increase in the removal efficiency of organic pollutants under visible light is mainly due to the strong π-π interactions between the pollutants and the graphene network, which contributes to the adsorption and degradation of the pollutants. In addition, the degradation of different antibiotics and dyes by SCN/GR under full-spectrum solar irradiation was also tested (Figure 7c). The degradation efficiencies of SCN/GR were 79%, 61%, 98%, and 59% for TC, SMX, RhB, and MO, respectively, which demonstrated the excellent photocatalytic performance of SCN/GR.

**Figure 8:**
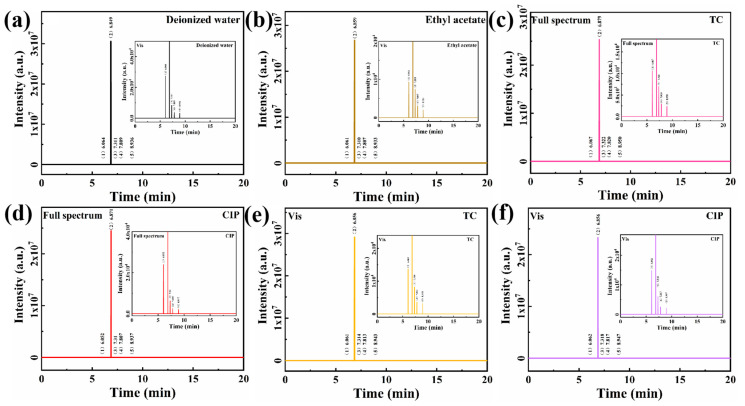
Gas chromatograms of (**a**) water and (**b**) ethyl acetate; Full spectrum of (**c**) TC and (**d**) CIP after degradation in the presence of SCN/GR. Visible light of (**e**) TC and (**f**) CIP after degradation in the presence of SCN/GR.

The degree of removal of organic pollutants can be demonstrated by photocatalytic degradation, but it does not prove that the pollutants are completely degraded into small molecules such as CO_2_ and H_2_O. Hence, the small molecule products were determined by gas chromatography, after full-spectrum solar and visible-light photocatalytic degradation, respectively (Figure 8). Following 60 min of degradation, the GC profiles of TC and CIP were analogous to that of ethyl acetate, with no other small molecules detected. The results indicated that SCN/GR was able to deeply decompose the pollutants into H_2_O and CO_2_. In conclusion, the excellent photocatalytic degradation ability of SCN/GR suggests that it has a promising future in antibiotic wastewater treatment.

### 3.4. Stability of SCN/GR

**Figure 9:**
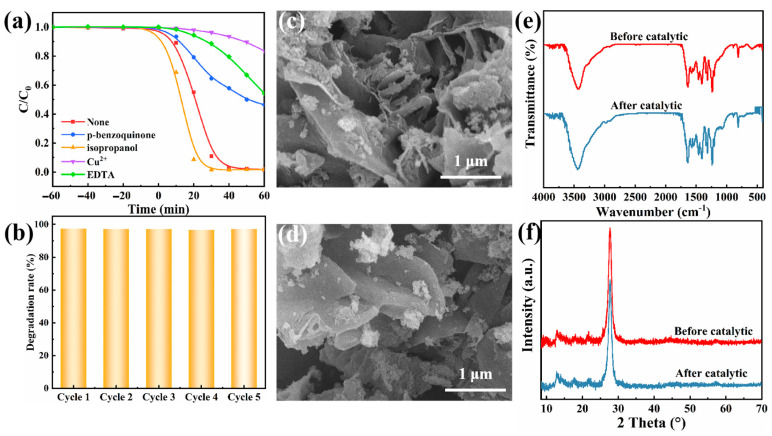
(**a**) Photocatalytic efficiency of SCN/GR after adding a quencher. (**b**) Photocatalytic recycle experiment of SCN/GR. (**c**) The microstructure of SCN/GR before photodegradation. (**d**) The microstructure of SCN/GR after photodegradation. (**e**) FT-IR spectra of the SCN/GR before photocatalysis. (**f**) XRD pattern of the SCN/GR after photocatalysis.

In order to distinguish the key role of active species on pollutant degradation efficiency, EDTA, Cu^2+^, benzoquinone, and isopropanol were used as scavengers for bursting h^+^, e^−^, ·O^2−^ and ·OH. Meanwhile, blank experiments were conducted for comparison. As shown in Figure 9a, the degradation efficiencies of RhB without scavengers, isopropanol, benzoquinone, Cu^2+^ and EDTA were about 98.2%, 98.2%, 53.6%, 16.5% and 45.7%, respectively. It is noteworthy that the introduction of isopropanol exerted little influence on the degradation efficiency, suggesting that ·OH had almost no effect on the photocatalytic performance. The degradation was significantly inhibited by the addition of benzoquinone, Cu^2+^ and EDTA, especially Cu^2+^. From this, it is tentatively concluded that ·O^2−^, e^−^ and h^+^ all play important roles in RhB degradation, and e^−^ is more active. This is due to the fact that e^−^ can react with O_2_ to generate ·O^2−^ in the photocatalytic system during the reaction process of SCN/GR. Therefore e^−^ is the source of ·O^2−^ generation, and the addition of Cu^2+^ affects both e^−^ and ·O^2−^.

The reusability of SCN/GR was tested as shown in Figure 9b. After five cycles, the photocatalytic degradation efficiency of SCN/GR remained above 98%, indicating that the SCN/GR photocatalyst has good stability. In order to investigate whether the morphology and structure of the photocatalyst changed before and after use, we performed SEM, FT-IR, and XRD tests (Figure 9c–f). The results showed that the SEM, FT-IR, and XRD of the composites did not change significantly before and after use, indicating that the morphology and crystal structure of SCN/GR did not change after the photocatalytic reaction, which is the main reason for its good recyclability and stability. Its excellent stability makes it reusable, which greatly improves the utilization of the photocatalyst in practical applications and has good potential utility in water environment remediation.

### 3.5. Photocatalytic Mechanism of SCN/GR

**Figure 10:**
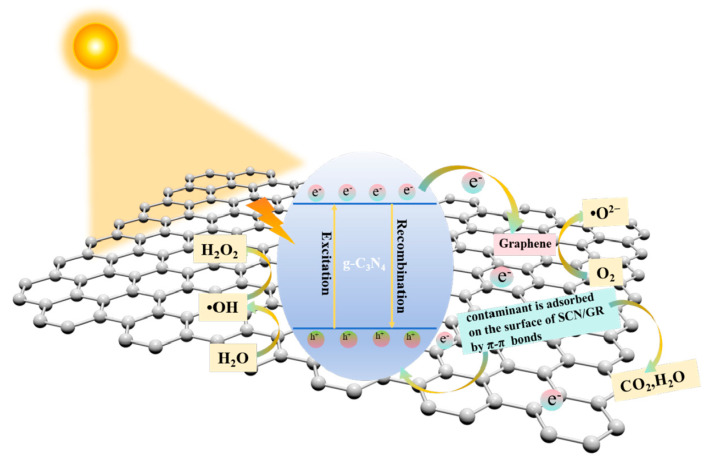
The proposed mechanism for photocatalytic degradation of pollutants over the surface of SCN/GR under illumination with sunlight.

The mechanism accounting for the improved photocatalytic performance of SCN/GR is put forward. To begin with, graphene nanosheets expanded the specific surface area of the photocatalyst and created additional active sites, which proved conducive to the adsorption of substances and the photocatalytic degradation process. Secondly, the composite of SCN and rGO not only provides more carrier transport paths, but also prolongs the life time of free electrons-this in turn creates more opportunities for electrons to take part in the pollutant degradation process. The photocatalytic mechanism of SCN/GR can be elaborated as follows: (1) enhanced absorption of visible light; (2) light-driven separation of photoinduced carriers and their transfer to the photocatalyst surface. (3) carrier-pollutant reaction occurs. As shown in Figure 10, under light irradiation, the charge on the valence band of SCN jumps to the conduction band and is subsequently transferred to rGO. rGO provides abundant charge transfer channels, and the photogenerated charge transferred to rGO not only directly participates in the reaction, but also promotes the formation of reactive radicals, which indirectly participate in the elimination of pollutants. In addition, the generated holes can directly participate in the removal of pollutants. Therefore, the introduction of rGO effectively inhibited the electron-hole pair complexation and provided more free carriers, thus enhancing the photocatalytic efficiency. The main reaction steps of the photocatalytic pollutant degradation mechanism under visible light are summarized in the following equation:

SCN + hv → SCN (e^−^) + SCN (h^+^) (1)

SCN (e^−^) + rGO → SCN + rGO (e^−^) (2)

SCN (e^−^) + O_2_ → ·O^2−^ (3)

rGO (e^−^) + O_2_ → ·O^2−^ (4)

Contaminant + ·O^2−^ + h^+^ + e^−^ → H_2_O + CO_2_ (5)

## 4. Conclusions

In conclusion, we have successfully fabricated 2D/2D g-C_3_N_4_/rGO (SCN/GR) hybrid photocatalysts. These hybrid materials exhibit significantly enhanced photocatalytic oxidation activity for antibiotics and dyes under visible light irradiation, compared to their pristine carbon nitride counterparts. The SCN/GR photocatalyst effectively reduces electron-hole pair recombination and enhances the migration efficiency of photogenerated carriers. The incorporation of rGO not only enhances the adsorption of pollutants on the SCN/GR surface but also improves the absorption of visible light, thereby potentiating the degradation of contaminants under visible light conditions. Moreover, the SCN/GR catalyst demonstrates the most superior performance among the synthesized samples, achieving a photocatalytic degradation efficiency of approximately 80% for antibiotics within a mere 1 h. Our findings underscore the high efficiency of the SCN/GR catalyst in the degradation of antibiotics via photocatalysis. This work offers an efficient and eco-friendly photocatalyst for the decomposition of organic pollutants and contributes to the advancement of sustainable remediation strategies. Furthermore, the SCN/GR hybrid photocatalyst showed high efficiency in the photocatalytic degradation of antibiotics, highlighting its potential for environmental remediation applications.

## Data Availability

The data are provided in manuscript and Supporting Information.

## Supplementary Materials

The following supporting information can be downloaded at: https://www.mdpi.com/article/10.3390/nano15211641/s1, Figure S1: (a) Electrochemical impedance diagram of SCN/GR. (b) LSV curves; Table S1: Analysis of specific surface area and pore size of samples.
