# Supplementary material for: In Situ Construction of 2D/2D g-C3N4/rGO Hybrid Photocatalysts for Efficient Ciprofloxacin Degradation
Source: Nanomaterials (Basel). 2025 Oct 28;15(21):1641. doi: 10.3390/nano15211641 (PMC12608163; doi:10.3390/nano15211641)
Supplement: Supplementary file 1 [file nanomaterials-15-01641-s001.zip › nanomaterials-3935246-supplementary.pdf]

Supporting Information

In Situ Construction of 2D/2D g-C_3N_4/rGO Hybrid Photocatalysts for Efficient Ciprofloxacin Degradation

Mengyao Wang[a], Yong Li[ab*], Rui, Li[a], Yali Zhang[a], Deyun Yue[a], Shihao Zhao[a], Maosong Chen[a], Haojie Song[ab*]

[a] School of Materials Science & Engineering, Shaanxi University of Science & Technology, Xi' an, 710021, PR China

[b]Shaanxi Key Laboratory of Green Preparation and Functionalization for Inorganic Materials, Shaanxi Laboratory of Advanced Materials.

[*] Corresponding author.
E-mail address: yongli@sust.edu.cn; songhaojie@sust.edu.cn

Materials

The following reagents are of analytical grade and utilized as received without further treatment. dicyandiamide, NH_4Cl and GO were purchased from eryer (Shanghai) Chemical Technology Co., Ltd (Shanghai, China).

Characterization

Samples were subjected to XRD analysis using a Smart Lab 9kW X-ray diffractometer. (Rigaku Corporation, Tokyo,Japan).SEM images were obtained on a Hitachi (SU8100) scanning electron microscope (Hitachi Ltd., Tokyo, Japan).TEM micrographs were taken on a JEOL (JEM-2100 F) transmission electron microscope (JEOL Ltd., Tokyo, Japan).X-ray photoelectron spectra (XPS) of the samples were collected using an AXIS SUPRA spectrometer. (Shimadzu Corporation, Tokyo, Japan).UV-visible-NIR absorption spectra were recorded with a Cary 5000 spectrophotometer.(Agilent Technologies Inc, Santa Clara, USA).

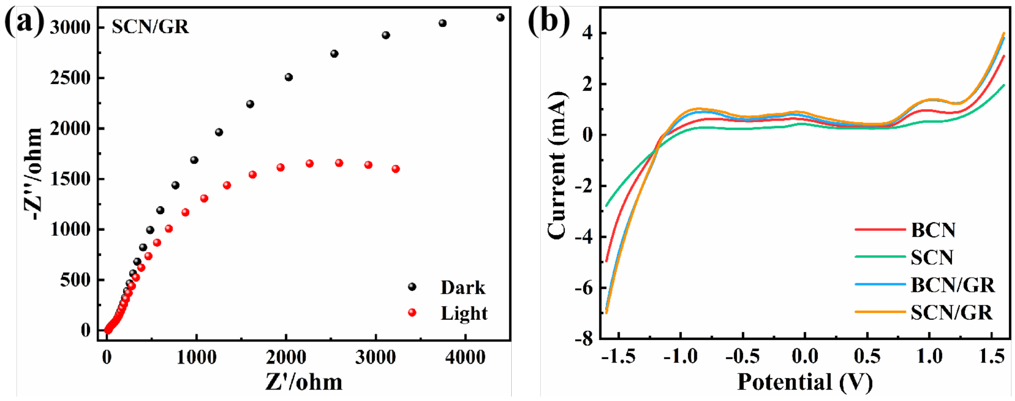

Fig. S1. (a) Electrochemical impedance diagram of SCN/GR. (b) LSV curves.

Table S1. Analysis of specific surface area and pore size of samples

Samples	as,BET[m^2g^{-1}]	Vm[cm^3(STP)g^{-1}]	Mean pore diameter[nm]
BCN	7.89	0.09	46.46
SCN	40.56	0.35	33.37
BCN/GR	13.38	0.12	42.47
SCN/GR	56.41	0.51	38.07
